# A Pilot Program to Promote Maternal and Infant Oral Health Through Collaboration Between Dental and Obstetric Providers: Impact on Dental Visits During Pregnancy

**DOI:** 10.1007/s10995-025-04158-8

**Published:** 2025-09-11

**Authors:** Sarah J. Clark, Divesh Byrappagari, Lindsay Sailor

**Affiliations:** 1 Susan B. Meister Child Health Evaluation and Research (CHEAR) Center, University of Michigan Health, 300 North Ingalls Street, Ann Arbor, MI 48109-5456 USA; 2 University of Detroit Mercy School of Dentistry, Detroit, MI USA; 3 Michigan Primary Care Association, Lansing, MI USA

**Keywords:** Pregnancy, Medicaid, Dental care, Perinatal oral health, Federally qualified health center (FQHC)

## Abstract

**Objectives:**

The Michigan Initiative for Maternal and Infant Oral Health (MIMIOH) program provided funding to federally qualified health centers (FQHCs) to hire and embed a dental hygienist in the obstetrics clinic, with the goal of increasing the provision of dental care during pregnancy. Ten sites participated in two cohorts. Our objective was to assess the impact of the MIMIOH program on receipt of dental visits during pregnancy.

**Methods:**

Using Medicaid paid claims, we documented quarterly trends in dental visits during pregnancy, describing trends for each MIMIOH sites and comparing aggregate results for MIMIOH Cohorts 1 and 2 vs. FQHCs that did not participate in MIMIOH.

**Results:**

The proportion of women with a dental visit during pregnancy varied across MIMIOH sites, and was higher during periods of active participation. For both MIMIOH Cohorts 1 and 2, the aggregate proportions of dental visits during periods of active participation was higher than that for non-MIMIOH FQHCs. In contrast, dental visits were lower for Cohort 1 after MIMIOH participation ended, and for Cohort 2 before MIMIOH began.

**Conclusions:**

The MIMIOH program was successful at increasing dental visits among pregnant women during periods of active participation.

## Significance

Despite prior research demonstrating the importance of perinatal dental care on oral health for women and their children, most pregnant women do not receive dental care during pregnancy, particularly low-income pregnant women. To address a component of access to dental services for low-income women, this study assessed the impact of a pilot integrated medical-dental program within federally qualified health centers on receipt of dental visits during pregnancy.

## Introduction

The perinatal period plays a vital role in the well-being and health of the mother and infant and a healthy mouth is essential for a healthy pregnancy for both mother and child (Committee on Health Care for Underserved Women, 2013). Research has shown that mother’s oral health has significant impact on the oral health of the infant (Shearer et al., 2011). Women with untreated dental caries are more likely to pass on cariogenic bacteria to their children leading to an increased risk for early childhood caries (Berkowitz, 2006; Chaffee et al., 2014; Xiao et al., 2019; Yost & Li, 2008).

Despite the importance of oral health during pregnancy, most pregnant women do not receive dental care during pregnancy, particularly low-income pregnant women (Centers 2022; MDHHS, n.d.; Lee et al., 2022). One factor is that many low-income women have pregnancy-associated Medicaid coverage, which covers medical and dental care during pregnancy and the immediate postpartum period but leaves gaps in coverage outside of the pregnancy-related coverage (Puett et al., 2022). The utilization of dental care during pregnancy also is influenced by workforce preparedness and willingness to provide care (ASTDD, 2020; Morgan et al., 2009). Although dental care during pregnancy has been deemed safe and necessary, many dental providers are still reluctant to provide care to pregnant women due to a lack of awareness and understanding of current guidelines, or concerns about liability (Lee et al., 2010; Pa Costa, et al., 2010). Denying or postponing care until after pregnancy can potentially cause harm if dental and periodontal conditions worsen. While many obstetrics & gynecology (OBGYN) residency programs in the US report including prenatal oral health training, many residents do not discuss oral health issues with their patients, due to time constraints, limited knowledge about oral health, or uncertainty about where to refer women for dental care (Curtis et al., 2013; Horowitz et al., 2019).

The separation of oral health and medical care delivery systems in the United States also contributes to disparities in access to dental services for underserved populations, exacerbated by a lack of infrastructure, technology, and personnel to help connect these separate systems (Northridge et al., 2020). Efforts to facilitate dental visits during pregnancy often focus on having prenatal care providers refer pregnant women for dental care. Some prior studies demonstrated that such referrals led to higher rates of dental visits during pregnancy (Russell et al., 2021), while others found that many women referred for dental care did not complete recommended treatments during pregnancy (Puett et al., 2022).

An evidence-based strategy to increase the use of dental care is the medical-dental integration model, which involves coordinating and integrating oral health services into a clinical setting such as primary care or behavioral health (APHA, 2020). This approach aims to improve access, quality, and outcomes of oral health care for patients, especially those who are underserved or at high risk. Embedding a dental hygienist into a primary care setting has shown some success in reducing health disparities, providing preventive care, coordinating care and referrals, and improving oral health outcomes for patients (Braun & Cusick, 2016; Linden et al., 2023). Medical-dental integration in an obstetric clinic may improve utilization of dental care for pregnant women. Both healthcare providers and pregnant women themselves have recognized the advantages of an integrated approach and expressed a desire for perinatal oral health care to be incorporated into prenatal care (Adeniyi et al., 2021; Adeniyi, Donnelly, Janssen, Jevitt, Kardeh Adeniyi et al., 2021a, b).

Federally Qualified Health Centers (FQHCs) are community-based healthcare providers that receive federal funding to offer a range of medical, dental, and behavioral health services to individuals regardless of their ability to pay (NACHC & DentaQuest Partnership for Oral Health Advancement, 2020). FQHCs play a pivotal role in providing comprehensive healthcare services to underserved communities. By offering dental services as part of their holistic approach to healthcare, FQHCs represent an attractive setting to attempt medical-dental integration focused on perinatal women. Prior work found that Medicaid beneficiaries in Michigan who received prenatal care at an FQHC had higher rates of dental visits during pregnancy (Byrappagari et al., 2024).

The Michigan Initiative for Maternal and Infant Oral Health (MIMIOH) is a program that promotes oral health and dental visits during pregnancy through close collaboration between dental and obstetric providers at FQHC clinics.

### Program Description

The University of Detroit Mercy School of Dentistry (SOD) was funded by the Michigan Department of Health and Human Services (MDHHS) Medicaid Division and Delta Dental Foundation to develop a pilot program to improve access to dental care for pregnant Medicaid-enrolled women. The pilot program, called the Michigan Initiative for Maternal and Infant Oral Health (MIMIOH), was developed by the School of Dentistry in partnership with the Michigan Primary Care Association (MPCA), which represents the state’s community health centers. The initial aim of the project was to examine the feasibility and impact of placing a registered dental hygienist within an OBGYN medical clinic.

The MPCA released a request for proposals inviting FQHCs to participate in the pilot project. To be eligible, FQHCs had to meet certain criteria, including having space within an OBGYN clinic to set up a dental operatory and providing prenatal care to at least 200 women per year. If selected, FQHCs would need to register as a PA-161 Public Dental Prevention Program, a designation that allows collaborative practice with indirect supervision of dental hygienists by dentists to provide preventive oral health care for underserved people. Under this designation, selected FQHCs would receive 100% funding for setting up the dental operatory and hiring a dental hygienist for the project’s first year. The dental hygienist would coordinate with the OBGYN staff to provide oral health education and assessment during prenatal visits and schedule dental visits with the on-site dental clinic. The objective was for the dental hygienist to meet with every eligible patient during each trimester of pregnancy.

Ten FQHCs participated in MIMIOH, comprising one site per FQHC. Cohort 1 initiated MIMIOH activities between October 2017 and July 2018 and continued through at least September 2019, although three sites experienced interruptions to their MIMIOH activities. Cohort 2 initiated MIMIOH activities between July and December 2019 and continued through December 2020; all experienced disruptions to their MIMIOH activities due to the COVID public health emergency. Table 1 presents a timeline of participation by cohort and site.

**Table 1:**
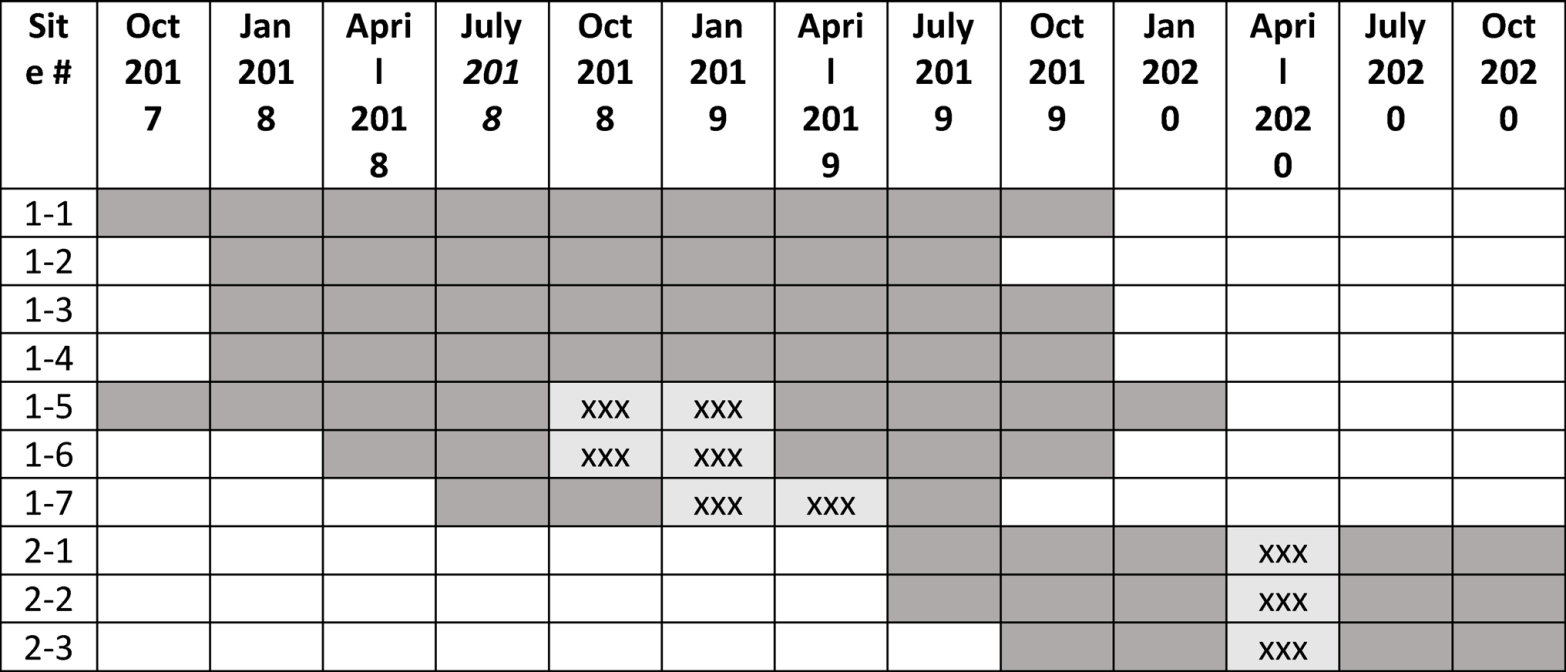
Timeline of MIMIOH participation, by cohort and site

Shading represents active participation; xxx represents a temporary interruption in program activity

The SOD Project Director and state coordinator, supported by MPCA, and MDHHS Oral Health Program staff, conducted in-person trainings for dental and OBGYN providers and staff at each MIMIOH site. The training included suggested protocols for setting up designated space and time for the dental hygienist within the OBGYN clinic; billing and recordkeeping, with a focus on clarifying which services were “billable” for the dental hygienist; and recommended processes to refer patients to the site’s dental clinic. Subsequently, SOD regional coordinators made monthly visits to the participating MIMIOH sites to offer ongoing technical assistance and address any concerns, and to gather monthly progress reports and program management information. In addition, SOD Project Director held monthly virtual meetings with MIMIOH site providers, administrators, and key personnel to receive updates, discuss any issues with the project’s implementation, and explore potential solutions.

To evaluate the impact of the MIMIOH program, we documented receipt of dental visits during pregnancy among women receiving prenatal care at MIMIOH sits vs. FQHCs that did not participate in the pilot project (“non-MIMIOH FQHCs”). Institutional Review Boards at the University of Michigan Medical School and the Michigan Department of Health and Human Services approved the study.

## Methods

### Study Population and Data Source

Our study population was women receiving prenatal care at an FQHC during the MIMIOH program period. Our data source was Michigan Medicaid enrollment files, which contain historical enrollment information, and administrative claims, which include service-level data on paid claims (fee-for-service) and encounters (managed care) with accompanying billing and reimbursement information.

We included all live birth deliveries between April 1, 2018, and December 31, 2020. We defined pregnancy as the 9 months prior to delivery, which encompasses the period from July 1, 2017, to December 30, 2020. We extracted data from the state Enterprise Data Warehouse in September 2021, which allowed ≥ 9 months for claims processing.

Consistent with an established quality measure (NCQA, n.d.), we identified delivery dates from claims with a delivery procedure or diagnosis code. We required a visit with a live birth diagnosis code within 30 days of the delivery date to exclude deliveries resulting in miscarriage or stillbirth. We considered delivery dates > 280 days apart to be separate deliveries. We documented the month and year of each live birth delivery.

For each delivery, we identified prenatal care visits as a bundled service or a visit for prenatal care or a prenatal visit with a pregnancy-related diagnosis code that occurred in the 9 months before delivery date. We counted each unique prenatal care service date as a prenatal care visit. We used billing codes to identify prenatal visits that occurred at an FQHC. We excluded deliveries with no prenatal care and with prenatal care only at non-FQHC sites.

We used enrollment files to document Medicaid coverage during the 9 months before delivery and we excluded those with less than 6 months of Medicaid coverage. The six-month minimum would ensure at least two trimesters of pregnancy for hygienist interactions and dental visits to occur.

**Fig. 1:**
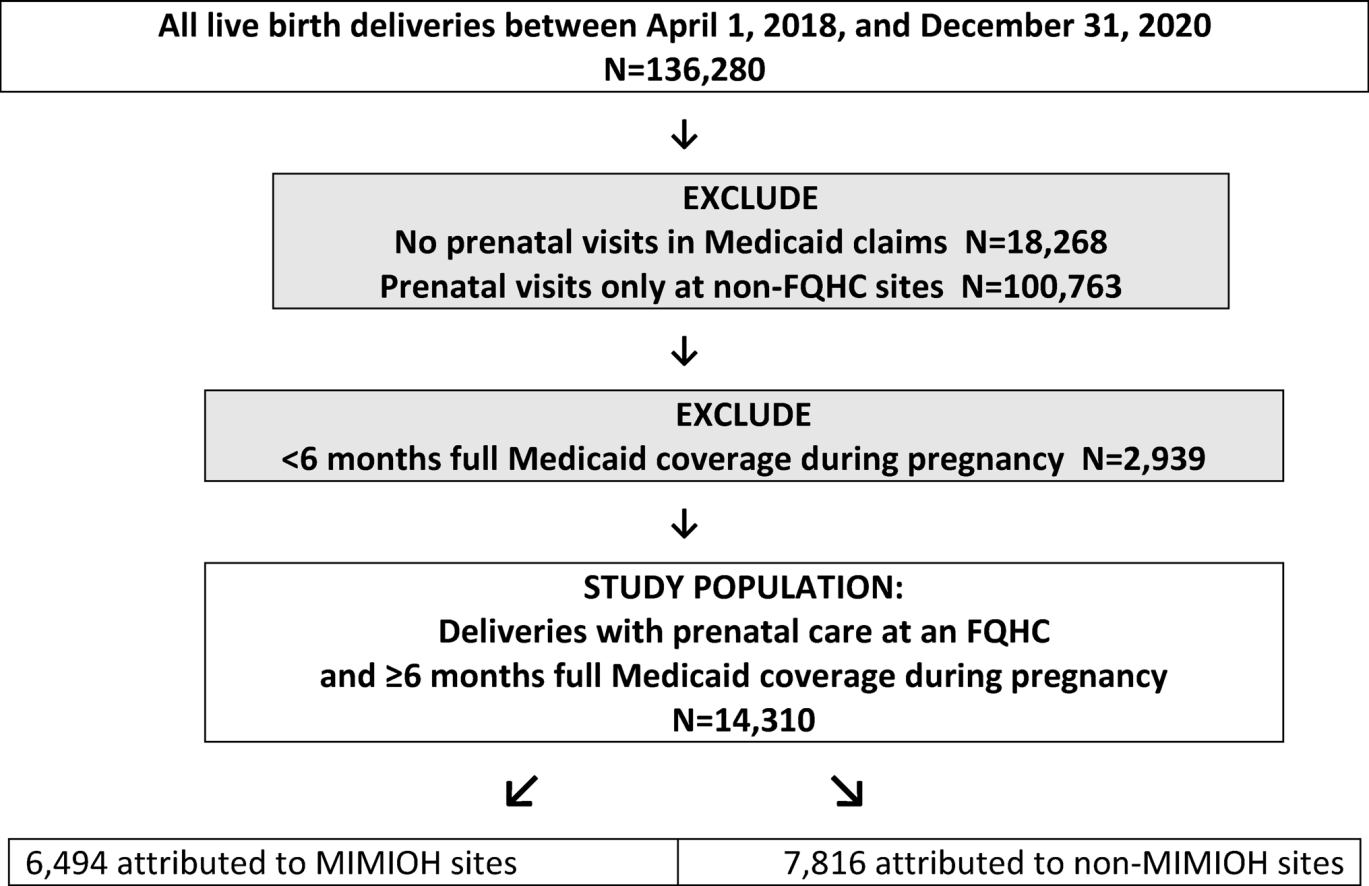
Flow diagram of study population identification

For the remaining deliveries, we assigned each prenatal care visit as occurring at one of the 10 MIMIOH sites or at any of 30 FQHCs not participating in MIMIOH. We attributed each delivery to a specific MIMIOH site or the comparison group of non-participating FQHCs based on the site with the most prenatal visits. Figure 1 presents a flow diagram of our process to identify the study population.

The study population included 14,310 deliveries with at least one prenatal care visit at an FQHC, including 6,494 deliveries with a prenatal care visit at a MIMIOH site. The number of deliveries at each MIMIOH site ranged from 223 to 1,049 across the study period (Table 2).

**Table 2:** Number of attributed deliveries, by cohort and site

	# of deliveries attributed to site (Apr 2018–Dec 2020)	
MIMIOH Cohort 1	5334	
1–1		1049
1–2		1016
1–3		876
1–4		792
1–5		976
1–6		223
1–7		402
MIMIOH Cohort 2	1160	
2 − 1		511
2–2		303
2–3		346
Non-MIMIOH FQHC	7816	

Our primary outcome was dental visits, defined as a paid claim with any procedure code in the form Dxxx and an outpatient setting of care. We calculated this outcome in two ways: first, we included dental visits in the 9 months before delivery (“during pregnancy”); second, we included dental visits in the 6 months before delivery, to represent on the period where interactions with the dental hygienist would be most likely to occur.

### Analysis

For each quarter from April 2018 to December 2020, we calculated the proportion of deliveries that had evidence of at least one dental visits during the prior 9 months and the prior 6 months. We documented these proportions for each MIMIOH site, aggregate for MIMIOH Cohort 1 and MIMIOH Cohort 2, and for non-MIMIOH FQHCs. For each timepoint, we used Fisher’s Exact Test to compare the aggregate proportion with dental visits for each MMIOH cohort vs. the non-MIMIOH FQHCs.

## Results

**Fig. 2:**
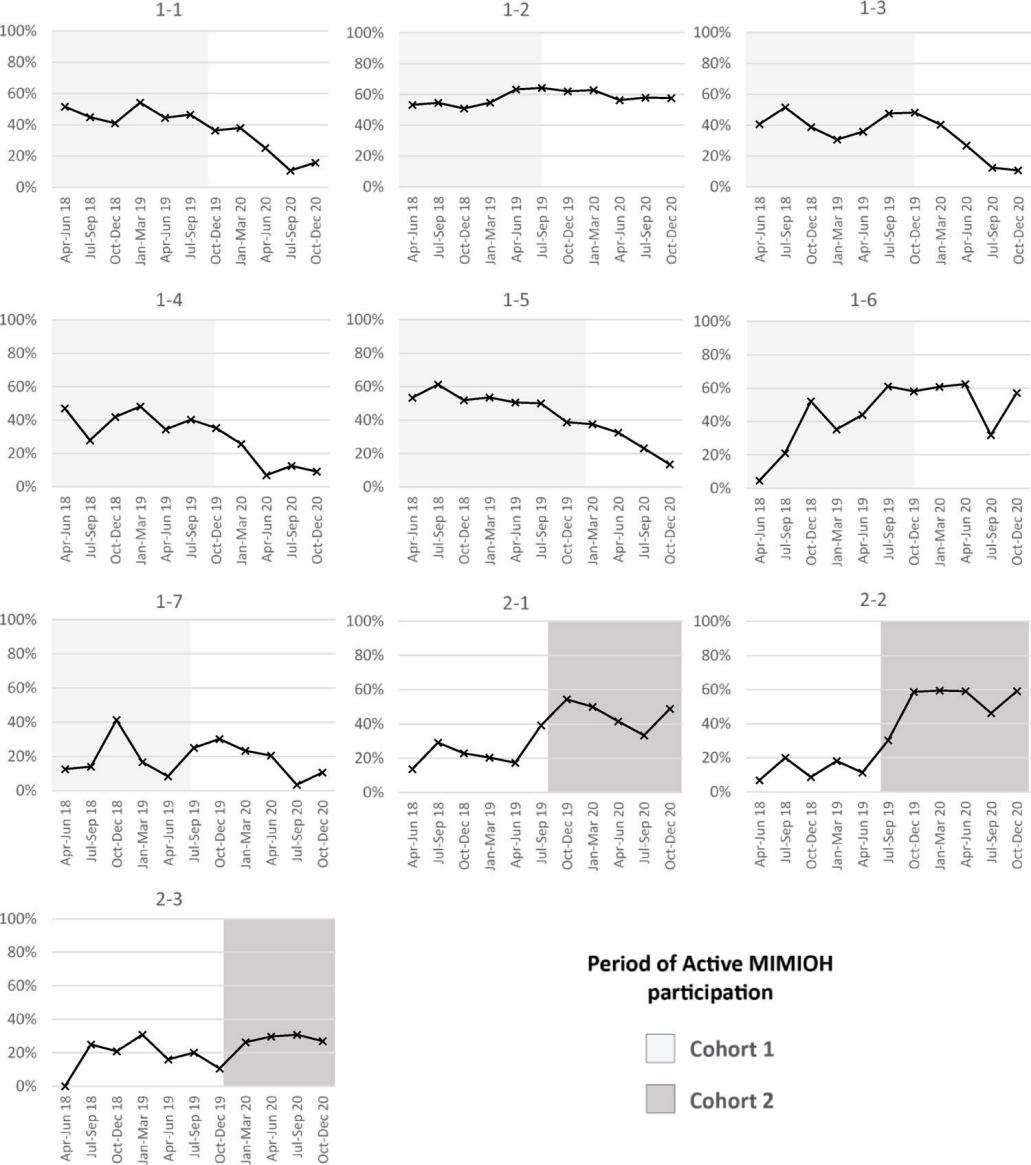
Quarterly trends in dental visits during pregnancy, by MIMIOH site

Figure 2 presents the trends in dental visits for each MIMIOH site; quarterly data are based on the month of delivery and reflect dental visits in the preceding 6 months. Five Cohort 1 sites followed the pattern of higher rates during program participation, with some drop-off afterward, concurrent with the COVID public health emergency; however, two Cohort 1 sites maintained relatively high visit rates. All Cohort 2 sites followed the overall Cohort 2 pattern of low rates initially, followed by increased visit rates after initiating the program; however, the magnitude of increase varied. These patterns were similar to those observed for dental visits in the 9 months prior to delivery (data not shown).

Table 3 compares the same quarterly trends in dental visits in the 6 months prior to delivery in aggregate for MIMIOH Cohort 1 and MIMIOH Cohort 2, compared to non-MIMIOH FQHCs. Across the study period, non-MIMIOH FQHCs demonstrated a relatively consistent proportion of dental visits, declining in later quarters of 2020, concurrent with the COVID public health emergency. In comparison, MIMIOH Cohort 1 demonstrated a significantly higher proportion of deliveries with a dental visit in the prior 6 months in all but the final quarter of 2020; however, the proportion with a dental visit decreased in the quarters after active MIMIOH participation ended.

**Table 3:**
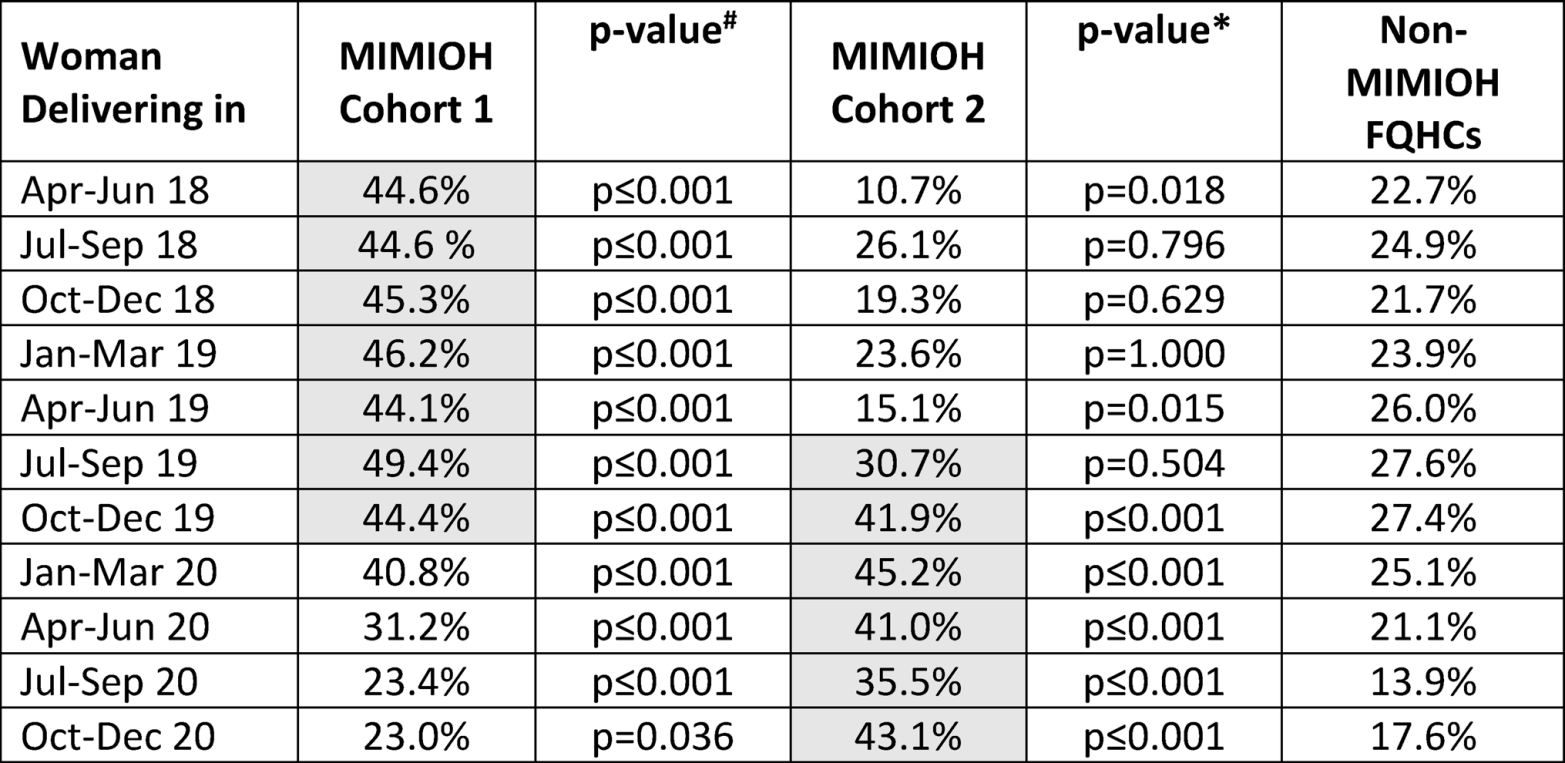
Proportion of women with a dental visit in the 6 months prior to delivery

Shading indicates period of active MIMIOH participation

At the outset of the study period, the proportion of deliveries with a dental visit during pregnancy was lower for MIMIOH Cohort 2 compared to non-MIMIOH FQHCs. Cohort 2 rates increased concurrent with initiation of MIMIOH participation and remained significantly higher than the compared to non-MIMIOH FQHCs even during the COVID public health emergency.

These patterns were similar to those observed for dental visits in the 9 months prior to delivery (data not shown).

## Discussion

The secondary analysis of Medicaid data demonstrates MIMIOH’s success in promoting prenatal dental care. During periods of active program participation, both MIMIOH cohorts demonstrated higher proportions of dental visits during pregnancy than the comparison group of non-participating FQHCs.

Within these overall patterns are unique trends for each MIMIOH site, which reflect the real-world challenge of cross-disciplinary integration. Regular interactions with MIMIOH site personnel, through both monthly virtual calls and the site visits with the regional coordinators, revealed common challenges across all sites. A key challenge was finding a hygienist able to maintain a flexible workflow, adjusting to the schedules and preferences of both OBGYN and dental providers, and able to create positive relationships with patients that would encourage them to receive the oral health advice and services. Other common challenges involved gaining buy-in from obstetricians and findings ways to convince them to emphasize the importance of dental care to their pregnant patients. Staff at each participating MIMIOH site had to learn these lessons as they worked toward implementation; some sites had a more challenging time than others. Some sites encountered specific barriers, including administrative decisions that limited the hygienist’s time in the obstetric clinic and physical distance between the medical and dental clinics. A characteristic of the most successful sites was presenting the dental visit as a routine part of obstetric care.

Financial sustainability was a substantial challenge for the MIMIOH sites. The PA-161 Program allows the hygienist to operate autonomously under indirect dentist supervision. To generate revenue commensurate with a hygienist’s salary and fringe benefits, MPCA calculated that the hygienist should aim to generate roughly 46 billable encounters per month. This estimate is based on Michigan Medicaid’s reimbursement rates under the health center’s Prospective Payment System (PPS), which covers only certain types of services. For MIMIOH, hygienists spent much of their time in patient education and assessments, but only prophylaxis and sealants qualified for reimbursement. Several sites tried to enhance financial sustainability by sending the dental hygienist to the pediatric clinic where they could provide reimbursable services to children. However, this meant time away from the OBGYN clinic and missed opportunities to interact with pregnant patients. For the many FQHCs with a limited number of pregnant patients, dental-OBGYN integration may not be sustainable without supplemental funding. We recommend that future initiatives construct realistic financial models based on which hygienist services are reimbursable and the volume of eligible patients.

Our findings are consistent with prior research in that the promotion of oral health in obstetric clinics can yield increased rates of dental visits during pregnancy (Russell et al., 2021). The MIMIOH goal was to have the hygienist interact with each pregnant patient at least once per trimester, though that was not always realistic in the context of clinic flow. Still, many women do not follow through to get needed dental care (Puett et al., 2022) even when clinic staff try to facilitate it. MIMIOH hygienists noted that they can emphasize the importance of having the dental visit, schedule the appointment, and even do a “warm hand-off” to the dental clinic but it is up to the individual to attend the scheduled visit.

### Study Strengths and Limitations

Unlike prior evaluations of programs promoting oral health for pregnant women (Puett et al., 2022; Russell et al., 2021), we used objective documentation of dental visits paid by Medicaid as our main outcome. These paid visits would include billable services performed by the dentist hygienist, but would not include non-billable services, such as oral health education or referral to dental care; as such, results do not reflect all interactions between the dental hygienist and pregnant patients.

Our primary outcome was receipt of any dental visit during pregnancy; results were consistent for 6 months and 9 months before delivery. We did not quantify the total number of dental visits received during pregnancy; nor did we characterize the type of dental visit, procedures performed, or location of dental visits.

Our study period did not include a pre-participation period for Cohort 1, so we could not determine whether the initiation of MIMIOH was associated with increased dental visit rates.

The COVID pandemic created significant constraints on dental care. For many sites, COVID impacted their typical patterns of dental care and affected their ability to continue MIMIOH participation. An unexpectedly positive finding was that most Cohort 2 sites increased rates of dental visits among pregnant women, despite dealing with COVID constraints during their period of active participation. In contrast, most Cohort 1 sites demonstrated a downward trend around the time their program participation ended and the COVID pandemic began; we cannot determine the extent to which the COVID pandemic altered that trajectory.

The MIMIOH program was based in FQHCs and is not generalizable to other settings. Deliveries not covered by Medicaid are not reflected in these data; FQHCs also serve uninsured and private-pay patients. Our results are not generalizable to all states. Throughout the study period Michigan included dental coverage for children and adults in its full scope of Medicaid plans and has a Medicaid expansion benefit to cover women outside of pregnancy. Thus, low-income women in Michigan likely have more extensive coverage for dental services before, during, and after pregnancy than their counterparts in other states with more limited policies (Puett et al., 2022).

Overall, the Michigan Initiative for Maternal and Infant Oral Health was successful in increasing rates of dental care among pregnant women during periods of active program participation. However, dental visit trends varied across participating sites, and increased dental visit rates were not sustained after program participation ended. Financial considerations impact the sustainability of medical-dental integration aimed at pregnant women.

## Data Availability

Available upon request.

## Code Availability

Available upon request.

## References

[CR1] Adeniyi, A., Donnelly, L., Janssen, P., Jevitt, C., Kardeh, B., von Bergmann, H., & Brondani, M. (2021a). Pregnant women’s perspectives on integrating preventive oral health in prenatal care. *Bmc Pregnancy And Childbirth*. 10.1186/S12884-021-03750-4 33794806 10.1186/s12884-021-03750-4 PMC8016156

[CR2] Adeniyi, A., Donnelly, L., Janssen, P., Jevitt, C., von Bergman, H., & Brondani, M. (2021b). A qualitative study of health care providers’ views on integrating oral health into prenatal care. *JDR Clin Transl Res*, *6*(4), 409–419. 10.1177/2380084420961998 10.1177/2380084420961998 32996370

[CR3] American Public Health Association (APHA) (2020). Improving access to dental care for pregnant women through education, integration of health services, insurance coverage, an appropriate dental workforce, and research. https://www.apha.org/policies-and-advocacy/public-health-policy-statements/policy-database/2021/01/12/improving-access-to-dental-care-for-pregnant-women. Accessed 12 July 2024.

[CR4] Association of State and Territorial Dental Direcors (ASTDD) (2020). Perinatal oral health policy statement. https://www.astdd.org/docs/perinatal-oral-health-policy-statement-2-26-2020.pdf. Accessed 12 July 2024.

[CR5] Berkowitz, R. J. (2006). Mutans streptococci: Acquisition and transmission. *Pediatric Dentistry*, *28*(2), 106–109. 16708784

[CR6] Braun, P. A., & Cusick, A. (2016). Collaboration between medical providers and dental hygienists in pediatric health care. *The Journal of Evidence-Based Dental Practice*, *16 Suppl*, 59–67. 10.1016/J.JEBDP.2016.01.017 27236997 10.1016/j.jebdp.2016.01.017

[CR7] Byrappagari, D., Cohn, L., Sailor, L., & Clark, S. J. (2024). Association between dental visits during pregnancy and setting for prenatal care. *Journal of Public Health Dentistry*, *84*(1), 21–27. doi.org/10.1111.jphd.12596 38173182 10.1111/jphd.12596

[CR8] Centers for Disease Control (CDC) (2022). Prevalence of selected maternal and child health indicators for michigan, pregnancy risk assessment monitoring system (PRAMS), 2016–2020. https://www.cdc.gov/prams/prams-data/mch-indicators/states/pdf/2020/Michigan-PRAMS-MCH-Indicators-508.pdf. Accessed 12 July 2024.

[CR9] Chaffee, B. W., Gansky, S. A., Weintraub, J. A., Featherstone, J. D. B., & Ramos-Gomez, F. J. (2014). Maternal oral bacterial levels predict early childhood caries development. *Journal of Dental Research*, *93*(3), 238–244. 10.1177/0022034513517713 24356441 10.1177/0022034513517713 PMC3929977

[CR10] Committee on Health Care for Underserved Women. (2013). Committee opinion 569: Oral health care during pregnancy and through the lifespan. *Obstetrics and Gynecology*, *122*(2 Pt 1), 417–422. 10.1097/01.aog.0000433007.16843.10 23969828 10.1097/01.AOG.0000433007.16843.10

[CR11] Curtis, M., Silk, H. J., & Savageau, J. A. (2013). Prenatal oral health education in U.S. dental schools and obstetrics and gynecology residencies. *Journal of Dental Education,**77*(11), 1461–1468. 24192411

[CR12] Horowitz, A. M., Child, W., & Maybury, C. (2019). Obstetric providers’ role in prenatal oral health counseling and referral. *American Journal of Health Behavior*, *43*(6), 1162–1170. 10.5993/AJHB.43.6.13 31662174 10.5993/AJHB.43.6.13

[CR13] Lee, H., Tranby, E., & Shi, L. (2022). Dental visits during pregnancy: Pregnancy risk assessment monitoring system analysis 2012–2015. *JDR Clinical & Translational Research,**7*(4), 379–388. 10.1177/23800844211028541 34323108 10.1177/23800844211028541

[CR14] Lee, R. S. Y., Milgrom, P., Huebner, C. E., & Conrad, D. A. (2010). Dentists’ perceptions of barriers to providing dental care to pregnant women. *Womens Health Issues*, *20*(5), 359–365. 10.1016/J.WHI.2010.05.007 20800772 10.1016/j.whi.2010.05.007 PMC2932670

[CR15] Linden, J. E., Gundacker, C. L. U., Deinhammer, L., & Crespin, M. (2023). Medical dental integration in wisconsin: Integrating dental hygienists into pediatric well child visits and prenatal care. *J Dent Hyg JDH*, *97*(3), 13–20. 37280104

[CR16] Michigan Department of Health and Human Services (MDHHS) Oral Health Dashboard: MI Mom’s Mouth-Michigan Medicaid Perinatal Oral Health Care Utilization, 2018–2021. https://www.michigan.gov/mdhhs/keep-mi-healthy/communicablediseases/epidemiology/chronicepi/oral-health-epidemiology/oral-health-dashboard_michigan-medicaid-perinatal-oral-health-care-utilization_2018-2021. Accessed 12 July 2024.

[CR17] Morgan, M. A., Crall, J., Goldenberg, R. L., & Schulkin, J. (2009). Oral health during pregnancy. *J Matern Neonatal Med*, *22*(9), 733–739. 10.1080/14767050902926954 10.3109/14767050902926954 19488943

[CR18] National Association of Community Health Centers (NACHC) & DentaQuest Partnership for Oral Health Advancement (2020). Oral health value-based care: the federally qualified health center (FQHC) Story. Boston, MA. 10.35565/DQP.2020.2013

[CR19] National Committee on Quality Assurance (NCQA) (2024). HEDIS measures and technical resources: prenatal and postpartum care (PPC). https://www.ncqa.org/hedis/measures/prenatal-and-postpartum-care-ppc/. Accessed 12 2024

[CR20] Northridge, M. E., Kumar, A., & Kaur, R. (2020). Disparities in access to oral health care. *Annual Review of Public Health*, *41*, 513–535. 10.1146/ANNUREV-PUBLHEALTH-040119-094318 10.1146/annurev-publhealth-040119-094318 PMC7125002 31900100

[CR21] Pa Costa, E. P., Lee, J. Y., Rozler, R. G., & Zeldin, L. (2010). Dental care for pregnant women: an assessment of North Carolina general dentists. *J Am Dent Assoc,**141*(8), 986–994. 10.14219/JADA.ARCHIVE.2010.0312 20675424 10.14219/jada.archive.2010.0312

[CR22] Puett, S., Tellez, M., Byrd, G., Weintraub, J. A., Ciszek, B., Phillips, C., Boggess, K., & Quinonez, R. (2022). Retrospective study of prenatal and postnatal gaps in oral health care utilization: Medicaid policy implications. *Maternal and Child Health Journal*, *26*(3), 642–648. 10.1007/S10995-021-03343-9 34997435 10.1007/s10995-021-03343-9

[CR23] Russell, S. L., Kerpen, S. J., Rabin, J. M., Burakoff, R. P., Yang, C., & Huang, S. S. (2021). A successful dental care referral program for low-income pregnant women in New York. *International Journal of Environmental Research and Public Health,**18*(23), 12724. 10.3390/ijerph182312724 34886450 10.3390/ijerph182312724 PMC8656616

[CR24] Shearer, D. M., Thomson, W. M., Broadbent, J. M., & Poulton, R. (2011). Maternal oral health predicts their children’s caries experience in adulthood. *Journal of Dental Research*, *90*(5), 672–677. 10.1177/0022034510393349 21248361 10.1177/0022034510393349 PMC3144114

[CR25] Xiao, J., Alkhers, N., Kopycka-Kedzierawski, D. T., Billings, R. J., Wu, T. T., Castillo, D. A., Rasubala, L., Malmstrom, H., Ren, Y., & Eliav, E. (2019). Prenatal oral health care and early childhood caries prevention: A systematic review and meta-analysis. *Caries Research*, *53*(4), 411–421. 10.1159/000495187 30630167 10.1159/000495187 PMC6554051

[CR26] Yost, J., & Li, Y. (2008). Promoting oral health from birth through childhood: Prevention of early childhood caries. *MCN; American Journal of Maternal Child Nursing,**33*(1), 17–23. 10.1097/01.NMC.0000305652.01743.8D 18158522 10.1097/01.NMC.0000305652.01743.8d

